# BCL-2 proteins: melanoma lives on the edge

**DOI:** 10.18632/oncoscience.193

**Published:** 2015-08-11

**Authors:** Jerry Edward Chipuk

**Affiliations:** Department of Oncological Sciences, The Tisch Cancer Institute, Icahn School of Medicine at Mount Sinai, New York, New York, USA

**Keywords:** BCL-2 family, chemotherapy, mitochondria, melanoma, oncogenic RAS/BRAF

Melanoma is potentially a fatal skin malignancy with more than 80,000 new cases in the US annually resulting in nearly 10,000 deaths (http://www.cancer.gov). Mutations within the MAPK pathway are *bona fide* oncogenes that are mutated in ~80% of melanoma cases [1,2]. The MAPK pathway consists of a series of proteins that link a growth factor (*e.g.,* EGF) activated plasma membrane receptor tyrosine kinase (*e.g.,* EGFR) to pro-survival/pro-proliferation transcriptional responses within the nucleus. In between the plasma membrane and the transcriptional responses are a series of adaptor proteins (*e.g.,* GRB2), a small GTPase (*i.e.,* RAS), and kinases (*e.g.,* RAF, MEK, ERK) that enable activation of the pathway via the phosphorylation of potent transcription factors [3]. In a broad range of melanomas, this pathway is specifically mutated at distinct steps (*e.g.,* RAS^G12V^ and BRAF^V600E^) leading to constitutive hyper-activation of pro-survival/pro-proliferation signaling in the absence of extracellular ligands [1,2]. As such, significant efforts have focused on the identification of small molecules that inhibit oncogenic MAPK signaling, including PLX4032 (Vemurafenib; BRAF^V600E^ inhibitor) and GSK1120212 (Trametinib; MEK inhibitor) [4].

Despite recent progress in the development and approval of targeted cancer therapies for RAS and BRAF mutant melanomas, improvements to progression free survival and overall survival remain an issue. The goal of inhibiting oncogenic MAPK signaling is to prevent cancer cell proliferation and ultimately induce apoptosis, a programmed form of cell death. Apoptosis is controlled by the availability of anti-apoptotic BCL-2 proteins (*e.g.,* BCL-2,), which reside at the outer mitochondrial membrane (OMM) [5]. Once the anti-apoptotic BCL-2 proteins are functionally inhibited by pro-apoptotic BH3-only proteins of the BCL-2 family (*e.g.,* PUMA), the OMM is compromised by the pro-apoptotic effectors BAK/BAX. These proteins form homo-oligomers within the OMM, leading to cytochrome *c* release and commitment to apoptosis. The goal of our recent work was to explore the utility of inhibitors to the anti-apoptotic BCL-2 proteins in lowering the threshold to PLX4032- and GSK1120212-induced apoptosis in melanoma cell lines [6].

We examined two “BH3 mimetics” in our study: ABT737 and ABT263 —-small molecules that function like BH3-only proteins to inhibit anti-apoptotic BCL-2 proteins and sensitize cancer cells to apoptosis [7]. While these molecules are in clinical trials for both liquid and solid tumors, minimal information was known about their potential in naïve melanoma cells (*i.e.,* never treated with targeted therapies) and drug resistant populations. Indeed, the combination of PLX4032 (or GSK1120212) and ABT737 (or ABT263) promoted rapid and robust apoptosis that was defined by BCL-2 family regulated cytochrome *c* release and caspase activation in naïve melanoma cell lines [6].

However, in PLX4032-resistant populations defined by no loss of phosphorylated ERK or G1 arrest after PLX4032 treatment, we noted a surprising effect of ABT737 with both conventional and targeted therapies [6]. PLX4032-resistant cell lines also demonstrated broad protection from multiple chemotherapies including: cisplatin, dacarbazine, and vinblastine. This suggested that PLX4032 resistance could occur through general targeting of the core apoptotic signaling components rather than exclusive deregulation of a drug-specific pathway. Curiously, the addition of ABT737 to PLX4032-resistant melanoma cells was sufficient to restore apoptotic responses to the drugs listed above, along with PLX4032; and the combination of PLX4032 and ABT263 prevented human melanoma tumor proliferation in a murine xenograft system by inducing marked apoptosis [6,8].

These results revealed an understudied concept in cancer biology that is while drug resistant populations may demonstrate negligible responses to conventional and targeted therapies, as measured by changes to pathway activation makers like phosphorylated ERK and/or cell cycle, covert activation of pro-apoptotic signaling may remain intact. This could provide a pharmacological opportunity to reduce tumor burden following certain therapeutic regimens. Of course, the addition of BH3 mimetics to targeted therapies may also reduce the likelihood of developing drug resistance if enhanced primary responses can be achieved. For ABT263, minimal side effects occur following its use as a single agent, therefore it may be an ideal candidate for combination strategies with PLX4032 and/or GSK1120212.

An alternative perspective to this argument is that targeted therapies in combination with BH3 mimetics may induce apoptosis that is not directly related to oncogenic pathway inhibition. This concept deserves investigation because we have noted that high concentrations of PLX4032 are required for ABT737 to reveal a pro-apoptotic effect in PLX4032-resistant cell lines, yet no changes to phosphorylated ERK or cell cycle are observable. Perhaps at high PLX4032 concentrations, additional off-target pro-apoptotic signaling occurs that cannot be functionally revealed or dissected in naïve melanoma cell lines.

**Figure 1:**
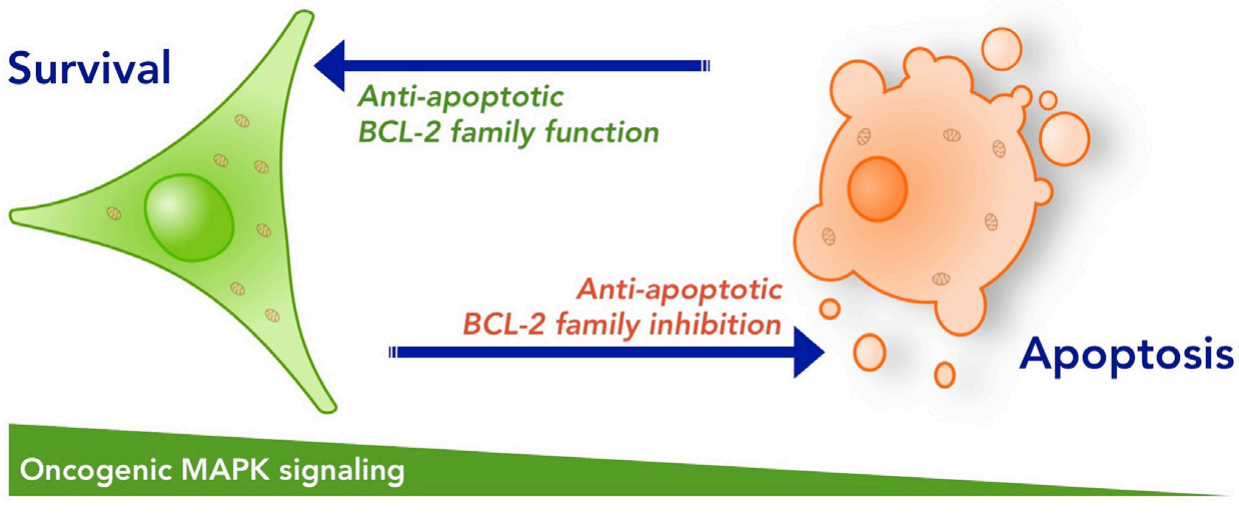
Anti-apoptotic BCL-2 proteins regulate cellular survival and apoptosis The anti-apoptotic BCL-2 proteins are responsible for maintaining cellular survival during developmental stages, and are required for cells to persist through stress conditions. Small molecule inhibitors to the anti-apoptotic BCL-2 proteins sensitize the majority of cells to apoptosis, but most frequently this is not sufficient to kill cancer cells. Collateral inhibition of oncogenic MAPK signaling and anti-apoptotic BCL-2 family function promotes marked apoptosis and has potential to reduce the development of drug resistance.

What is certain is that we are at the beginning of understanding how BCL-2 proteins promote melanoma survival and apoptosis, and how these proteins may be pharmacologically targeted to increase primary treatment responses and perhaps treat drug resistant disease (Figure 1). Given the recent development of highly specific BH3 mimetics and additional small molecules targeting the pro-apoptotic BCL-2 proteins, we will continue exploring how melanoma cells can be pushed over the edge to their death.
